# Chronic Kidney Disease and Cellular Senescence

**DOI:** 10.3390/ijms27073205

**Published:** 2026-04-01

**Authors:** Marya Morevati, Juliette Tavenier, Morten Scheibye-Knudsen, Morten Baltzer Houlind, Aram Hedayati, Mads Hornum

**Affiliations:** 1 Department of Nephrology and Endocrinology, Rigshospitalet, University of Copenhagen, 2100 Copenhagen, Denmark; 2 Department of Clinical Research, Copenhagen University Hospital Amager & Hvidovre, 2650 Hvidovre, Denmark; 3 Institute of Cellular and Molecular Medicine, University of Copenhagen, 2200 Copenhagen, Denmark; 4 Independent Researcher, 1799 Copenhagen, Denmark

**Keywords:** cellular senescence, premature aging, chronic kidney disease, kidney fibrosis, senescence-associated secretory phenotype, senotherapy, artificial intelligence

## Abstract

Chronic kidney disease (CKD) and kidney aging share many pathological and molecular features, with cellular senescence emerging as a potentially important contributor to disease progression. Senescent cells accumulate in the kidneys due to persistent stressors, contributing to chronic inflammation and fibrosis via the senescence-associated secretory phenotype (SASP). This review explores the intersection between CKD and renal aging, focusing on the mechanisms driving senescence, its impact on kidney function, and potential therapeutic interventions. We explore various senotherapeutic approaches, such as senolytics, senomorphics, and rejuvenating agents, and highlight the increasing role of artificial intelligence (AI) and machine learning (ML) in detecting and monitoring senescent cells, enabling high-throughput and precise assessment across experimental and clinical settings. Understanding these mechanisms offers new avenues for developing targeted treatments to slow CKD progression and improve patient outcomes.

## 1. Introduction

Chronic kidney disease (CKD) is defined as a persistent loss of renal function and affects approximately 10–15% of the world’s population, making it a significant global public health issue [1,2,3]. Irrespective of the origin of CKD, renal fibrosis stands as a common hallmark, identifiable through histological features such as glomerulosclerosis, tubular atrophy, and interstitial fibrosis [4]. The progressive nature of CKD may lead to end-stage renal disease (ESRD), necessitating renal transplantation or dialysis [5,6].

CKD is defined by an abnormal estimated glomerular filtration rate (eGFR), structural changes in kidney histology and albuminuria and is especially prevalent in the elderly, with higher incidence and prevalence rates than in younger age groups [7,8,9]. Furthermore, accumulating evidence indicates a remarkable resemblance between the characteristics of CKD and the aging kidney [10,11]. Accordingly, CKD is often viewed as a state of accelerated renal aging [12]. The processes of aging and CKD also share numerous common triggers and underlying mechanisms, such as oxidative stress, inflammation, mitochondrial dysfunction, activation of the renin–angiotensin–aldosterone system (RAAS), and hyperactive Wnt/β-catenin and transforming growth factor beta (TGF-β) signaling [13,14]. Epigenetic modifications, including DNA methylation, histone acetylation, and methylation, are also implicated in kidney aging and CKD progression [15]. The accumulation of senescent cells in various renal compartments is increasingly recognized as a shared phenomenon of both premature aging and CKD, as evidenced in diverse animal models and human kidney biopsies [16,17].

**Figure 1:**
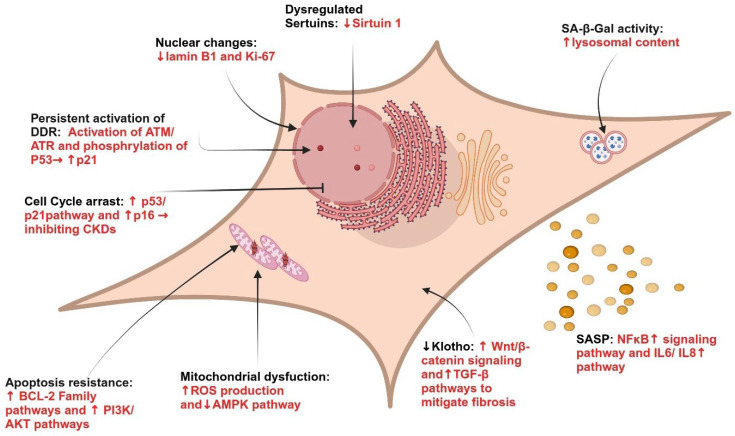
Hallmarks of senescence in renal cells. Cellular senescence is a permanent state of cell-cycle arrest characterized by the SASP, persistent activation of the DDR, phosphorylation of p53, upregulation of p21 and p16, and resistance to apoptosis. Senescent cells are larger than normal and exhibit an irregular shape. DNA damage in these cells leads to increased nuclear changes, γH2AX phosphorylation, and a reduction in sirtuin 1 and Lamin B1 levels. Additionally, senescent cells display elevated lysosomal activity (SA-β-Gal), endoplasmic reticulum stress, mitochondrial dysfunction, and decreased Klotho levels. Established hallmarks of cellular senescence include activation of the DDR, p53/p21-mediated cell-cycle arrest, SA-β-Gal activity, and γH2AX signaling. In contrast, metabolic alterations such as lipid metabolism disruption, glutamine catabolism, and NAD^+^ metabolism changes represent emerging associations that may contribute to senescence and CKD progression. Abbreviations: SASP: senescence-associated secretory phenotype; DDR: DNA damage response; SA-β-Gal: senescence-associated beta-galactosidase.

Cellular senescence is a key hallmark of aging and is characterized by irreversible cell cycle arrest (Figure 1) [18,19]. Cellular senescence is crucial in regulating various biological events in different contexts. Transient and acute cellular senescence is required during embryogenesis, tissue repair, and tumor suppression [20,21]. On the other hand, chronic and persistent senescence following injury results in the buildup of senescent cells and in the development of a pronounced and detrimental senescence-associated secretory phenotype (SASP) consisting of numerous proinflammatory and profibrotic factors, which contribute to chronic inflammation and tissue fibrosis [18,22]. The kidney is particularly susceptible to senescence-driven pathology due to several physiological characteristics. Renal tubular epithelial cells have high metabolic demand and rely heavily on mitochondrial ATP production, making them vulnerable to oxidative stress and mitochondrial dysfunction. In addition, the kidney is continuously exposed to filtered toxins and metabolic waste products, including protein-bound uremic toxins that accumulate during CKD and can induce DNA damage responses and inflammatory signaling. The renal microvasculature also operates close to hypoxic thresholds, and microvascular rarefaction in CKD further promotes hypoxia-induced stress. Finally, several renal cell populations exhibit limited regenerative capacity, including podocytes and certain tubular epithelial cells, which increases the likelihood that injury responses result in persistent senescence rather than effective regeneration.

Currently, no curative treatment exists for CKD. The existing treatment approaches mainly involve lifestyle and dietary adjustments and blood pressure management through the blockade of renin–angiotensin–aldosterone system (RAAS), sodium-glucose co-transporter-2 (SGLT2) inhibition, and optimization of glycaemic control, including the use of glucagon-like peptide-1 (GLP-1) receptor agonists and non-steroidal mineralocorticoid receptor antagonists (nsMRAs) [23,24,25,26,27,28,29,30,31,32]. Although these interventions primarily slow down CKD progression rather than completely block it, emerging evidence indicates that SGLT2 inhibitors, GLP-1 receptor agonists, and RAAS blockade may also modulate cellular aging and senescence pathways [23,24,25,26,27,28,29,31,33]. These observations suggest that current standard-of-care therapies for CKD may partly exert their protective effects through modulation of pathways linked to cellular senescence and biological aging. Given this situation, it becomes crucial and urgent to understand the underlying mechanisms of CKD and develop novel therapeutic strategies. This review investigates the intersection between CKD and kidney aging, highlighting their shared pathological features and focusing on cellular senescence. Furthermore, it explores potential approaches for targeting senescent cells as a viable strategy for developing therapeutic interventions for age-related kidney disorders.

## 2. CKD: A Condition of Renal Aging

The leading risk factors driving CKD vary according to the specific environment, with hypertension and diabetes being prevailing culprits. Additional factors, such as human immunodeficiency virus (HIV) and exposure to toxins or heavy metals, can also contribute to kidney pathology and are more prominent in developing nations [5]. Interestingly, there are areas of the world with notably high rates of CKD where the exact cause remains unknown or unclear [5]. Histopathologically, CKD is characterized by the activation of α-smooth muscle actin (α-SMA)-positive myofibroblasts, excessive production and accumulation of extracellular matrix, leading to tissue fibrosis, infiltration of inflammatory cells, tubular atrophy, and microvascular complications [4,34,35]. CKD complications include chronic inflammation, protein–energy wasting (PEW), muscle wasting and weakness, vascular calcification, osteoporosis, and cardiovascular disorders, which are all characteristic features of aging [36].

As individuals age, kidney function naturally declines, with the decrease in eGFR starting at around age 30 at a rate of 0.7–0.9 mL/min/1.73 m^2^ per year in healthy individuals [37]. With age, the kidney undergoes a series of changes that resemble those observed in CKD, including a reduction in the number and size of nephrons, glomerulosclerosis, tubular atrophy, inflammation, dyslipidemia, interstitial fibrosis, and an increase in the prevalence of vascular rarefaction and arteriosclerosis [38,39,40,41]. It is worth emphasizing that renal senescence can also manifest in children suffering from kidney disease, potentially contributing to a reduced capacity for renal regeneration [42].

Furthermore, aging kidneys, similarly to kidneys in CKD, are susceptible to injury, oxidative stress, inflammation, and fibrosis and often struggle to regenerate and recover [43,44,45]. However, these changes are typically milder during normal aging than in CKD. Therefore, in many ways, CKD may be likened to a state of premature or accelerated renal aging [44]. Premature aging is a condition where aging-like changes manifest early, often accompanied by multi-organ hypofunction, increased vulnerability to injury, and a high risk of diseases [44]. This resemblance partly reflects the unique physiological context of the kidney, where high metabolic activity, chronic exposure to circulating toxins, susceptibility to hypoxia, and limited regenerative capacity of key cell populations amplify stress responses and promote the accumulation of senescent cells. At the cellular level, typical features of premature aging include the accumulation of senescent cells and stem cell exhaustion [46,47]. Disruption or dysregulation of critical signaling pathways, such as DNA damage, oxidative stress, telomere shortening, loss of Klotho, and oncogene activation, can lead to premature aging [48,49]. Recent proteomic and transcriptomic studies provide evidence that cellular senescence contributes to CKD progression rather than solely reflecting aging [50]. CKD patients can be stratified into senescence-based endotypes (sendotypes), where a high-senescence signature dominated by TNF, NF-κB, and MAPK signaling is associated with worse renal function and faster eGFR decline [50]. These senescence-associated pathways were further validated in human CKD biopsies and kidney organoid injury models, confirming their involvement at the tissue level [50]. These findings suggest that CKD is biologically heterogeneous with respect to senescence signaling. The sendotype framework may therefore provide a basis for precision senotherapeutic strategies, where therapies targeting specific inflammatory or senescence pathways (e.g., NF-κB or MAPK signaling) could be applied to patient subgroups most likely to benefit.

Therefore, understanding the pathophysiological mechanisms underlying cellular senescence and premature aging is essential for elucidating the cause and mechanism of CKD and designing effective intervention strategies. Current evidence suggests a bidirectional relationship between kidney injury and cellular senescence, where stressors such as hypoxia, toxin accumulation, and metabolic dysfunction induce senescence, while senescent cells further promote inflammation and fibrosis through SASP signaling, thereby exacerbating kidney damage.

## 3. Type of Cellular Senescence

### 3.1. Primary Subtypes

Cellular senescence can be categorized into two primary subtypes: acute and chronic. Acute cellular senescence may be induced by extracellular signals targeting specific cell populations within the tissue. This orchestrated process can yield beneficial outcomes, including the modulation of embryogenesis, the containment of tumorigenesis, the facilitation of wound healing, and the enhancement of tissue restoration [51]. Acute senescence serves as a protective reaction in response to various kidney injuries, facilitating immune clearance and tissue restoration [52]. In a timely manner, infiltrating macrophages eliminate acutely senescent cells. However, if the immune system fails to keep up with the pace of senescent cell generation and senescent cells are not promptly eliminated, they tend to accumulate over time and have the potential to develop into chronic senescence [53]. In the kidney, this immune surveillance is partly mediated by natural killer cells, which recognize and eliminate senescent renal tubular epithelial cells through NKG2D-dependent activation and perforin-mediated cytotoxicity, a process that can be impaired under immunosuppressive conditions [54].

**Figure 2:**
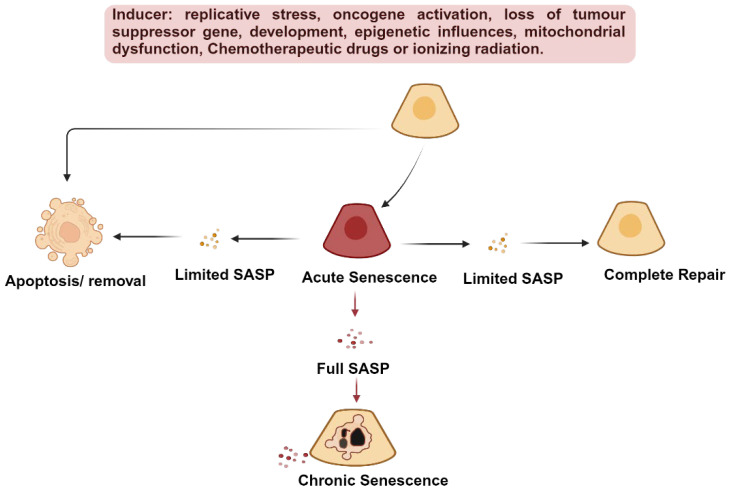
Cellular senescence in the kidney is a multifaceted process shaped by several triggers and pathways. Replicative stress occurs when cell proliferation shortens telomeres, hindering DNA replication and initiating the DDR, leading to replicative senescence. Oncogene activation can directly induce DDR or activate signaling pathways (e.g., MDM2-p53-p21 or p38AMPK-p16), resulting in cell cycle arrest, and cellular senescence. Loss of a tumor suppressor gene can trigger senescence via the Akt-mTOR-p53 pathway. During embryonic development, senescent cells play a role in shaping and remodeling various tissues (limbs, nervous system, gut endoderm, mesonephros), with p21 playing a significant role. Epigenetic alterations, including histone modifications and DNA methylation changes, stimulate senescence through the p16-RB pathway and transcriptional reprogramming during cellular senescence. Mitochondrial dysfunction leads to excessive reactive oxygen species production and oxidative stress, causing DNA damage and activating the DDR or ERK-p16-RB pathway. Chemotherapy drugs, chemicals, and ionizing radiation can induce DNA damage, triggering senescence. These various cellular senescence inducers can lead to acute or chronic cellular senescence. In cases where the trigger is sudden and intense and the SASP is limited, this may result in either apoptosis or complete removal and repair by immune cells. However, when the trigger is persistent and of lower intensity, the sustained SASP activation or the full SASP engagement may result in chronic senescence. Abbreviations: DDR: DNA damage response; SASP: Senescence-Associated Secretory Phenotype.

Additionally, prolonged exposure to stressors or persistent damage can also trigger chronic senescence, which plays a key role in hindering cell renewal and repopulation [55]. It also contributes to the progression of CKD by inducing chronic inflammation and fibrotic reactions through the release of SASP factors. Primary senescent cells originate following various detrimental stimuli, including oxidative or proteostatic stress, defects in DNA repair, activation of oncogenes, and the shortening of telomeres, among others (Figure 2). In the kidney, senescence may arise through distinct mechanisms, including replicative senescence driven by telomere attrition and stress-induced premature senescence (SIPS). In largely post-mitotic or slowly proliferating renal cells, such as podocytes, senescence is more likely driven by stressors including oxidative stress, hypoxia, and uremic toxins rather than replicative exhaustion. These mechanistic differences may influence both the selection of senescence markers and the response to senotherapeutic interventions. Each of these triggers can shape the phenotype of senescent cells, and similarly, the same trigger can result in different senescent phenotypes in cells from different cell types [56].

### 3.2. Secondary Senescence

The secretion of SASP factors by primary senescent cells can drive secondary senescence through two fundamental mechanisms [57]. The first mechanism, known as paracrine senescence, involves releasing SASP factors that propagate senescence both locally and systemically in the extracellular fluid and via the bloodstream [58]. The second mechanism, referred to as juxtacrine senescence, entails primary senescent cells inducing and reinforcing senescence in adjacent cells through direct cell-to-cell interactions, including cell fusion and intracellular protein transfer [59,60,61,62]. Consequently, secondary senescence can lead to a small subset of senescent cells expanding and spreading to distant locations, further contributing to age-related diseases.

A study has shown that the transplantation of relatively small quantities of senescent cells into the peritoneum of young mice is sufficient to induce enduring physical dysfunction and propagate cellular senescence within the host tissues. Furthermore, the transplantation of a smaller number of senescent cells yielded a similar impact in older recipients, leading to decreased survival [63]. This underscores the potency of senescent cells in diminishing both health and lifespan and indicates the potential for the spread of senescence-associated complications within different tissues.

## 4. Identifying Cellular Senescence

### 4.1. Biomarkers and Hallmarks of Cellular Senescence

Cellular senescence can be identified using a range of hallmarks and biomarkers, though no single marker consistently or reliably defines this complex state [64]. Senescent cells typically display an enlarged size, an increased activity of senescence-associated beta-galactosidase (SA-β-Gal), an accumulation of lipofuscin granules, and senescence-associated heterochromatin, among others [65,66]. Table 1 summarizes key markers frequently utilized to detect senescence, particularly in kidney research [17].

Among these, SA-β-Gal activity, linked to increased lysosomal content, is the most widely used marker [67,68]. However, relying solely on SA-β-Gal as a marker for senescent cells has limitations, as its activity can increase in non-senescent cells in dense cell cultures, and it is unsuitable for identifying senescent cells in paraffin-embedded tissue [67]. To address these limitations, researchers often examine additional molecular markers and pathways associated with senescence.

For example, signaling pathways leading to permanent cell cycle arrest, such as the p16/pRb and p53/p21 pathways, are critical. Early growth response 2 (Egr2), a transcriptional activator of these pathways, has been proposed as a potential senescence marker [69]. Other promising markers include tRNA-derived fragments, positive cofactor 4 (PC4), histone acetyltransferase KAT7, and Cdkn1a transcript variant 2 [70]. Elevated levels of Cyclin-Dependent Kinase (CDK) inhibitors such as p16^Ink4a^ (p16), p21^Cip1^ (p21), p19^ARF^ (in mice), p14^ARF^ (in humans), p27^KIP1^, and p15^INK4b^ are also indicative of senescence. DNA damage, another hallmark of senescence, is often marked by histone H2AX phosphorylation (γ-H2AX), which is associated with senescence-related alterations in DNA organization [71,72]. Additionally, nuclear changes, such as the accumulation of senescence-associated DNA damage foci (SADF) and senescence-associated heterochromatic foci (SAHF), reflect alterations in DNA structure and organization. Overexpression of SAHF-associated proteins induces senescence and represses genes related to cell proliferation [72].

Other changes include downregulation of cell proliferation markers such as Ki67 and PCNA as well as depletion of Lamin B1 (LMNB1), a structural nuclear protein [73]. Notably, the decrease in LMNB1 has been demonstrated in both apoptotic and senescent cells. However, the underlying cause for this reduction is different between these two cell states. Apoptotic cells experience LMNB1 degradation by caspases, whereas senescent cells exhibit reduced LMNB1 levels due to decreased mRNA stability [67]. Urinary clusterin has also been reported as a non-invasive biomarker of renal epithelial senescence and is associated with the progression of human kidney disease [74].

Additionally, single-cell RNA sequencing reveals that different senescent subtypes may exist, underscoring the need for a combination of markers for accurate determination of cellular senescence. A proposed three-step multi-marker workflow involves assessing SA-β-Gal activity as the first step, followed by co-staining for markers such as p16, p21, γ-H2AX, and LMNB1, and finally examining senescence markers relevant to the specific biological context [75].

**Table 1:** Markers commonly used to detect senescent cells in kidney. Abbreviations: FFPE: formalin-fixed, paraffin-embedded; CDK: cyclin-dependent kinase; pRb: retinoblastoma protein.

	Biomarker	Properties	Function and Outcome	Sample Type	Ref.
Upregulated in senescent cells	SA-β-Gal	Senescence-associated β-galactosidase	Reflects elevated lysosomal activity with aging in kidney	in vitro, Fresh, Frozen	[17,76,77]
	p16^Ink4a^	Cyclin-dependent kinase (CDK) inhibitor	Interacts with the CDK4 and CDK6 complex, leading to the inhibition of their activity, causing dephosphorylation of pRb, and subsequently suppressing the transition from G1 to S phase.	in vitro, Fresh, Frozen, FFPE	[78,79]
	p21^Cip1^	CDK inhibitor	Primarily blocks CDK2 prompting cell cycle arrest in G1/S phase.	in vitro, Fresh, Frozen, FFPE	[11,17,80,81]
	ARF	CDK inhibitor alternate reading frame	In humans as p14^ARF^ and in mice as p19^ARF^, also leads to cell cycle arrest at G1/S phase.	in vitro, Fresh, Frozen, FFPE	[77]
	γ-H2AX	The phosphorylated form of H2AX, and a selective marker of DNA double-strand breaks	The combination of high levels of γ-H2AX and the absence of Ki-67 serves as an indicator of DNA damage-induced senescence in kidney transplants. It can trigger pathways that induce cell cycle arrest through the DNA damage response (DDR).	in vitro, Fresh, Frozen, FFPE	[67,82,83]
	SAHF	Senescence-associated heterochromatin foci	Demonstrates modified DNA packaging in senescent cells.	in vitro, Fresh, Frozen, FFPE	[84,85]
Downregulated in senescent cells	Ki-67	A marker for actively proliferating cells	A nuclear protein linked to cell proliferation	in vitro, Fresh, Frozen, FFPE	[42]
	LMNB1	protein located in the nuclear lamina	Drives changes in nuclear morphology through a mechanism that relies on both p53 and Rb.	in vitro, Fresh, Frozen, FFPE	[67,78,86]

### 4.2. Artificial Intelligence and Machine Learning for Identifying Senescence in CKD

In kidney disease, artificial intelligence (AI) and machine learning (ML) are increasingly applied in two complementary areas: (i) omics-based biomarker discovery and patient stratification, and (ii) imaging-based detection of senescent cells in histological samples (Table 2). These approaches differ substantially in their translational readiness and clinical applications.

#### 4.2.1. Omics-Based Biomarker Discovery and Patient Stratification

Integrated ML-based bioinformatics approaches in CKD, including diabetic kidney disease, identified hub genes (LIMA1, ZFP36, FOS, IGFBP6, CKB) that were combined into a senescence-related risk biomarker for disease stratification [87]. Similarly, ML-based pipelines have highlighted PTEN as a protective gene in diabetic nephropathy and enabled the identification of circulating senescence-associated biomarkers CKAP4 and PTX3, which can identify high-risk patients for acute and CKD [88,89]. A broader perspective on this field emphasizes that AI can efficiently analyze high-dimensional omics data to uncover clinically relevant senescence biomarkers and support precision medicine [94].

#### 4.2.2. Imaging-Based Detection of Senescent Cells

AI has also advanced the imaging-based detection of senescent cells, which has long been limited by the case-specific nature of classical markers such as SA-β-Gal, p16^INK4a^, and p21^CIP1^. Recent methods exploit nuclear morphology, chromatin texture, and LMNB1 depletion to identify senescent cells from resting or DNA-damaged cells with high accuracy [90,91,92]. These approaches have enabled the generation of tissue senescence scores and have been applied in both cell culture and histological images to quantify the burden of senescent cells, evaluate senolytic responses, and provide reproducible, high-throughput monitoring strategies. AI-derived senescence scores may have several potential clinical applications in kidney disease. These include patient stratification in clinical trials of senotherapeutic therapies, identification of high-risk patients with accelerated biological aging, and monitoring of treatment responses. In the future, integration of imaging-based senescence detection with circulating biomarkers or urinary markers may enable less invasive assessment of renal biological aging.

Extensive research programs are laying the groundwork for spatial and population-scale mapping of senescence. The SenNet project, initiated by the US National Institutes of Health (NIH), utilizes state-of-the-art multiomics techniques and high-content spatial imaging to map senescent cells across human and mouse tissues, creating comprehensive datasets that enable the development of four-dimensional senescence atlases and will support future AI-assisted analytics [95,96,97]. Complementary work by Scheibye-Knudsen and colleagues has applied deep learning to identify senescent cells based on altered nuclear morphology in cell cultures, tissue micrographs and blood smears, and even to reveal how environmental factors such as high-altitude living influence aging progression [90,91,92,93]. By integrating traditional biomarkers with these cutting-edge technologies, researchers can establish robust and high-throughput approaches to detect and monitor senescent cells across various biological and pathological contexts. Despite these advances, several challenges remain for clinical translation of AI-based senescence detection. First, the absence of a single gold-standard marker for cellular senescence introduces ground-truth uncertainty, which may affect the reliability of supervised ML models. Second, domain shift between datasets, caused by differences in staining protocols, tissue preparation, scanner platforms, or imaging conditions, can substantially reduce model performance when applied across institutions. Finally, robust clinical deployment will require multi-center validation using harmonized biopsy cohorts linked to longitudinal clinical outcomes, such as eGFR decline, in order to establish reproducibility and regulatory readiness.

## 5. Mechanisms Behind Cellular Senescence

Although the precise mechanisms of cellular senescence remain only partially understood, numerous external stressors and stimuli that trigger senescence have been identified. These stressors operate through distinct mechanisms, ultimately leading to permanent cell cycle arrest.

### 5.1. Increased Expression of Cyclin-Dependent Kinase (CDK) Inhibitors

Cellular senescence entails a network of signaling pathways, with the p53/p21 and p16 pathways being the primary drivers. When stressors induce DNA damage, the ATM protein kinase and p53, p21, and p16 become activated as a response [98]. Ultimately, these occurrences inhibit CDK complex phosphorylation and the retinoblastoma protein (Rb), causing cell proliferation to halt and cellular senescence to occur. The p53/p21 pathway is thought to play a part in triggering cellular senescence, whereas p16 signaling is predominantly implicated in maintaining and initiating the senescent phenotype [17].

### 5.2. Senescence Maintenance via Senescent Cell Anti-Apoptotic Pathways (SCAPs)

SCAPs encompass mechanisms that are responsible for prolonging the survival of senescent cells, despite the presence of triggers that would typically lead to apoptosis, such as their own SASP [75]. SCAPs involve the activation of various factors such as the BCL-2 family, ephrin ligand B1 (EFNB1), EFNB3, Forkhead box O-4 (FOXO-4), HSP90/p-AKT, and p21/JNK, all of which play crucial roles in senescent cell survival and persistence [99,100].

Specifically, BCL-2 hinders autophagy by interacting with the autophagy protein Beclin1 and inhibiting the formation of autophagosomes [101]. FOXO-4 functions as a sequester for p53 in the nucleus, thereby restricting p53-mediated apoptosis [76]. Notably, interfering with the FOXO-4-p53 interaction leads to improved kidney function [76]. The activation of p21 prevents senescent cells from undergoing apoptosis by constraining JNK signaling and caspase activation [102]. Additionally, the stabilization of p-AKT by HSP90 contributes to the extended survival of senescent cells [103].

### 5.3. Reduced Expression of Age-Affecting Proteins SIRT1 and Klotho

Klotho and sirtuin 1 (SIRT1) are intrinsic elements investigated for their anti-aging properties and their potential to prevent cellular senescence and premature aging. Klotho is a single-pass transmembrane protein mainly present in the kidney; however, our group has also shown that Klotho is expressed in the liver of naked mole rats [104,105]. Klotho also acts as a co-receptor for fibroblast growth factor 23 (FGF23), a hormone involved in phosphate metabolism that is markedly elevated in CKD, and emerging evidence suggests that dysregulation of the FGF23–Klotho axis may contribute to oxidative stress, inflammation, and pathways linked to cellular senescence [104]. Research has shown that serum levels of Klotho decline with age and in CKD, implying its possible use as a biomarker of aging [104,106]. Mice lacking Klotho display an accelerated aging phenotype, encompassing a shorter lifespan, infertility, impaired growth, diminished cognitive function, and abnormal calcification [107]. Conversely, transgenic mice overexpressing Klotho demonstrate extended lifespan and protection against age-related disorders [108]. Klotho may exert its protective effects by inhibiting the p53/p21 and Wnt/β-catenin pathways [109,110]. Specifically, Klotho was shown to protect against CKD by safeguarding aging cells from mitochondrial dysfunction and cellular senescence through the inhibition of Wnt/β-catenin signaling [111].

SIRT1 is an NAD^+^ dependent deacetylase acting on both histone and nonhistone proteins like FOXO, p53, and NF-κB. Thus, SIRT1 significantly influences central signaling pathways linked to cellular senescence and aging [112]. SIRT1 is broadly expressed in regular renal tubular cells and podocytes, but its levels diminish with the onset of renal diseases or during aging [113]. Investigations suggest that the depletion of SIRT1 in podocytes of mice results in glomerular sclerosis, a sign of aging [114]. Accordingly, the elimination of SIRT1 in endothelial cells can induce senescence via p53 acetylation, whereas overexpression of SIRT1 prevents or reverses senescence by suppressing p53 activity [115,116,117]. Moreover, research indicates that sodium tetrasulfide (Na2S4) can directly sulfhydrate SIRT1, hindering p65 NF-κB and STAT3 phosphorylation/acetylation, thus alleviating diabetic renal lesions [118].

Senescent cells have also been implicated in the disruption of NAD^+^ metabolism by secreting SASP factors which can activate the NAD^+^ consuming enzyme CD38 in macrophages. This may result in increased NAD^+^ consumption in CD38-containing tissues and result in the depletion of NAD^+^ with age [119,120]. Our group has observed an overexpression of the CD38 gene 24 h after induction of severe acute kidney injury (AKI) in rats, and this overexpression persisted continuously for 14 days post-surgery. This occurred in conjunction with the overexpression of other proinflammatory markers, even though other parameters in this model were returning to normal [106]. These findings indicate that senescent cells contribute to a decline in the level of NAD^+^, which may drive senescence-dependent age-related conditions, e.g., diabetes [121].

### 5.4. Newly Identified Pathways in the Kidney and Senescence

Recent discoveries have highlighted several additional pathways associated with kidney aging and cellular senescence, offering potential targets for therapeutic intervention. Among these, NF-κB inhibitors play a central role in regulating senescence and aging processes in the kidney. By reducing oxidative DNA damage and cellular stress, these inhibitors delay the onset of senescence. This effect is mediated through the regulation of IKKε, IKKα, and IKKβ, which are influenced by proinflammatory cytokines, pathogens, oxidative stress, and growth factors [122]. Targeted deletion of Myd88, an adapter protein in the NF-κB signaling pathway, has further demonstrated its protective role in kidney health by reducing fibrosis and preventing the accumulation of senescent tubular epithelial cells following folic acid-induced kidney injury [123]. Myd88 deletion also reduced the expression of proinflammatory cytokines such as IL-1α, IL-1β, IL-6, TNF-α, and MCP-1, leading to improved outcomes in renal fibrosis. This suggests the involvement of innate immune signaling in cellular senescence after kidney injury [123].

In addition to intrinsic cellular pathways, immune cells may also contribute to the inflammatory milieu associated with senescence in CKD, as circulating monocytes and other immune cells can undergo functional reprogramming and promote chronic inflammatory signaling.

The Wnt/β-catenin signaling pathway has been identified as another critical contributor to age-related renal fibrosis and cellular senescence [103,104]. Activation of this pathway induces senescence, as indicated by increased levels of senescence markers like p16, p53, and p21, as well as enhanced SA-β-Gal activity in renal tubular epithelial cells [77]. In addition, the calcium-activated chloride channel accessory 1 (CLCA1), acting via the TMEM16A/Cl^−^ current pathway, has emerged as a newly recognized factor in aging-related kidney damage [124]. Interestingly, Benidipine exerts renoprotective and cardioprotective effects through the triple blockade of L-, N-, and T-type calcium channels. Moreover, this calcium channel blocker has been shown to promote the clearance of cigarette smoke-induced senescent cells and alleviate lung emphysema [125].

Another contributor to kidney aging is the cannabinoid receptor 2 (CB2), which has been linked to mitochondrial impairment in renal tubular cells. CB2 activation appears to accelerate mitochondrial dysfunction, negatively impacting renal aging and health [126].

Finally, circadian rhythm regulation also plays a pivotal role in regulating aging processes. Disruptions in circadian balance have been associated with increased senescence while maintaining proper circadian function supports cellular repair mechanisms [127]. Additionally, intestinal microbiome imbalances and microbial metabolism alterations have been shown to impair cellular reparative potential and contribute to aging processes [128,129,130,131].

## 6. Cellular Senescence in Renal Disease

Current evidence suggests that different renal cell populations, including tubular epithelial cells, podocytes, endothelial cells, and interstitial cells, may undergo senescence depending on the underlying disease trigger and renal compartment involved.

Considerable diversity has been observed in the characteristics of senescent cells, depending on the cell type and the triggers initiating senescence. Cellular senescence has been identified as a fundamental contributor to various disorders, including cardiovascular, hepatic, and renal diseases [132,133]. CKD exhibits numerous phenotypic resemblances to systemic aging within human subjects and animal models. While the proximal tubular epithelium is typically the primary site for senescent cell accumulation following kidney injury, senescence also develops in other areas, such as glomeruli and endothelium [13,134]. It is widely recognized that different stressors affecting distinct cell types can influence the distribution and characteristics of senescent cells. Table 3 provides a comprehensive overview of studies identifying senescent cells in kidney aging and various kidney disorders.

In humans, elevated renal levels of p16 expression and heightened SA-β-Gal activity have been documented across multiple stages of CKD and in diverse kidney conditions like IgA nephropathy, membranous nephropathy (MN), focal segmental glomerular sclerosis (FSGS), minimal change disease (MCD), and diabetic nephropathy (DN) [135]. Furthermore, investigations have unveiled an increased prevalence of senescent cells in various kidney compartments during aging and instances of kidney dysfunction [136]. Cell cycle arrest or senescence significantly influences the transition from AKI to CKD [137]. In kidney transplantation, aged kidneys often lead to poorer outcomes, with cellular senescence emerging as a critical determinant of graft survival [138]. A study involving 75 preimplantation renal allograft biopsies found a correlation between p16 expression levels and postoperative renal function in humans [139]. These findings highlight the potential of senescence biomarkers for risk stratification in kidney transplantation. Although transplantation and ischemia–reperfusion injury represent important clinical contexts for studying renal senescence, a detailed discussion of these topics is beyond the scope of this review, which primarily focuses on senescence mechanisms in CKD progression. Additionally, a retrospective clinical investigation revealed a connection between cellular senescence in the kidney tubular epithelia of individuals with IgA nephropathy (IgAN) and disease progression [80].

Mechanistic research involving p16-null mice has revealed that the absence of p16 enhances kidney regeneration and reduces capillary rarefaction post-ischemic injury [140]. Furthermore, utilizing transgene INK-ATTAC technology to selectively eliminate p16-positive senescent cells through drug intervention has shown the potential to alleviate age-related traits, including preservation of kidney function, as well as extend healthspan [141,142].

Consequently, reducing cellular senescence emerges as a promising therapeutic strategy [143]. While human studies primarily provide associative evidence, mechanistic animal models, including p16-null mice and INK-ATTAC transgenic systems, demonstrate that experimental removal of senescent cells can improve renal function and reduce fibrosis, supporting a potential causal role of senescence in CKD progression.

**Table 3:** Senescence-driven mechanisms and therapeutic targeting across kidney diseases.

Disease Context	Model	Dominant Senescent Compartment(s)	Dominant Senescence/SASP Axes	Functional Consequence	Best Therapeutic Hypothesis	Ref.
Aging Kidney	Human	Tubular epithelial cells; podocytes; interstitial/vascular cells	TGF-β, COX-1/COX-2 signaling	Impaired renewal, nephron loss, and renal fibrosis	Senolytics/senomorphics/immune-mediated clearance of senescent cells	[22,144,145]
Aging kidney	Mice and rats	Proximal tubules and glomerular podocytes	Cell-cycle arrest pathways (p16^INK4a^, p19^ARF^, p21^CIP1^) with SA-β-Gal activity	Reduced regenerative capacity	Senolytics/senomorphics	[142,146,147]
AKI	Multiple mouse models	Proximal tubular epithelial cells	Cell-cycle arrest pathways (p16^INK4a^, p21^CIP1^, p27^Kip1^), DNA damage signaling (γ-H2AX)	Maladaptive repair, fibrosis	Senolytics/senomorphics/immune-mediated clearance to enhance repair	[137,144,148]
IgA Nephropathy	Human	Renal tubular epithelial cells	Pro-fibrotic ECM signaling	Fibrosis progression	Senolytics/senomorphics	[80]
Diabetic nephropathy	Human	Tubular epithelial cells and podocytes	Hyperglycemia-associated inflammatory and profibrotic signaling	Tubulointerstitial fibrosis	Metabolic control + senotherapy	[134]
Diabetic nephropathy	Mouse STZ	Tubular epithelial cells	Hyperglycemia-driven inflammatory signaling	Tubular injury	Metabolic modulation (SGLT2i)	[81]
MN, FSGS	Human	Glomerular cells and interstitial cells	Proteinuria-associated pro-inflammatory and pro-fibrotic stress signaling	Glomerular dysfunction	Senomorphics	[149]
Fibrosis	Mouse (IRI UUO, ADR model)	Renal tubular epithelial cells	TGF-β1 profibrotic SASP axis (Wnt9a–TGF-β loop)	Interstitial fibrosis	Senomorphic	[77]
Renal transplantation	Human	Infiltrating cells in interstitial area, tubular epithelial cells, vascular cells	Pro-inflammatory cytokines and TGF-β signaling	Graft dysfunction	Senolytics/senomorphics/immune-mediated clearance to reduce senescent burden	[22,139]

## 7. Cellular Senescence in Renal Fibrosis

This section will focus on mechanisms that link senescence specifically to renal fibrogenesis and CKD progression, while emphasizing processes particularly relevant to fibrosis, including maladaptive tubular repair during the AKI-to-CKD transition and profibrotic epithelial–mesenchymal signaling. Renal fibrosis impacts all components of the kidney and is evident in various kidney diseases. It is commonly recognized as the primary factor influencing the gradual decline in renal function and the prognosis of CKD [150], and has a substantial impact on the transition from AKI to CKD, a common characteristic observed in ESRD [34,151,152]. Tubulointerstitial fibrosis is the predominant form of renal fibrosis, arising from the buildup of proteins in the extracellular matrix (e.g., the fibrillar collagens, fibronectin). Moreover, the inflammatory response of renal tubular epithelial cells has a central role in the glomerular, interstitial, and vascular segments, coinciding with a significant decline in GFR, and hinders the restoration of epithelial cells [34,153,154,155]. Mechanistically, persistent injury can lock proximal tubular epithelial cells in G2/M arrest, which promotes secretion of profibrotic mediators through JNK-dependent signaling and contributes to interstitial fibrosis.

We recently demonstrated the involvement of circulating endotrophin, a precursor of collagen VI, in the pathogenesis of AKI and its prognostic significance for mortality after AKI [156]. Furthermore, we identified its role in promoting fibrosis in cases of IgA nephritis and ANCA vasculitis [157]. Despite a comprehensive understanding of tubular pathologies, inflammation, infiltration of inflammatory cells, fibroblast activation and expansion, and compromised microvasculature being associated with renal fibrosis [34,106,151], the exact pathogenesis of this condition remains incompletely elucidated. Further research is needed to comprehensively untangle the mechanisms underlying the development of renal fibrosis and the contribution of senescent cells to this process.

Due to the gradual impairment of renal functional reserve, renal senescence likely enhances susceptibility to CKD [158]. In CKD, accumulation of protein-bound uremic toxins such as indoxyl sulfate and p-cresyl sulfate promotes renal cellular senescence, as these albumin-bound toxins are poorly cleared by dialysis and induce oxidative stress, DNA damage responses, mitochondrial dysfunction, cell-cycle arrest, and SASP signaling [159]. In fact, elevated levels of p16 and increased SA-β-Gal activity often precede renal alterations across various stages of CKD and CKD-related renal conditions [160]. Notably, several studies have demonstrated a correlation between senescence markers and the extent of kidney fibrosis in patients and animal models [98,161,162]. Lymphocytes of CKD patients overexpress p53, and mesenchymal stem cells derived from CKD-afflicted rats exhibit premature senescence [163,164]. Wnt/β-catenin signaling has also been linked to renal fibrogenesis partly through induction of senescence programs in tubular epithelial cells, including evidence for a role of Wnt-9a in experimental renal fibrosis [77].

Furthermore, in human transplanted kidneys undergoing the transition from AKI to CKD, there is a substantial up-regulation of p21 levels [137,165]. In an ischemia–reperfusion injury (IRI) model of CKD, elderly mice display more pronounced pathological changes in terms of renal fibrosis, inflammation, and microvascular rarefaction compared to the young control group [137]. Moreover, the absence of p21 has been shown to prevent fibrosis and alleviate the progression to CKD in mouse models [166]. These observations collectively imply that renal senescence plays a crucial part in the development and advancement of CKD. Mitochondrial dysfunction and impaired antioxidant signaling (including NRF2-related pathways) can reinforce senescence and fibrogenesis and are discussed in detail in Section 5.

Beyond classical profibrotic pathways, metabolic stress responses that affect tubular repair capacity may also modulate senescence-associated fibrogenesis. A recent study has also shown that FGF21 and autophagy work together to counteract kidney disease progression during aging [167]. FGF21 deficiency accelerates kidney aging in autophagy-deficient mice, with mRNA levels of p19 and immunostaining for p21 and phospho-H2AFX/γ-H2AX (H2A.X variant histone), indicating exacerbated cellular senescence in the proximal tubular epithelial cells of aged FGF21- and Atg5-deficient mice [167].

Despite these insights, the exact role of cellular senescence in renal fibrosis remains inadequately explored, highlighting the need for further investigation into its contribution to kidney disease progression.

## 8. Treatments for the Aging Kidney

Due to the significant role of cellular senescence in kidney diseases and the aging process, targeting senescent cells has become a potential therapeutic approach for treating CKD [168]. To target these conditions, a category of interventions known as senotherapeutics is being developed to address senescence [10]. Encouragingly, animal models with genetically reduced senescent cells have displayed reversed aging traits, improved recovery from kidney damage, enhanced organ function, and extended lifespan [169]. The existing strategies for senotherapy encompass a range of methodologies, including senolytics (compounds that selectively eliminate senescent cells), senomorphics (compounds that inhibit senescent characteristics such as the SASP), externally sourced cell-based products, and non-pharmacological therapies and rejuvenating agents [170,171]. The safety and efficacy of senotherapeutic interventions are also likely to vary across sendotypes and CKD stages, with earlier stages potentially offering a more favorable therapeutic window compared to advanced CKD or dialysis, where reduced regenerative capacity and increased frailty may limit benefit. Table 4 highlights key senotherapeutic strategies employed to address kidney-related issues arising from cellular senescence.

### 8.1. Senolytic Interventions

Senolytic interventions can be achieved by overcoming senescent cells’ resistance to apoptosis through interference with SCAP pathways activity, thereby inducing programmed cell death [140]. Senolytics, including quercetin, a natural flavonoid found in certain fruits and vegetables, eliminate senescent vascular smooth muscle and endothelial cells in animal models [172,208]. Quercetin activates various pathways, such as AMPK, SIRT1-PINK1-mediated mitophagy, and NRF2-NF-κB signaling, to induce apoptosis in senescent cells [172,208,209,210]. It has been demonstrated that quercetin reduced senescence markers in obese mice’s kidneys and positively affected cardiac function in high-fat diet-fed mice due to its pro-angiogenic activity [211]. Additionally, in an in vitro study, quercetin significantly reduced the expression of NAMPT at the protein level. Narita et al. showed in mice that NAD^+^ stimulates components in SASP through NAMPT, the enzyme of the NAD^+^ salvage pathway [212]. Quercetin inhibited nicotinamide mononucleotide (NMN), an NAD^+^ metabolite in the salvage pathway, accumulation in mesangial cells cultured under high glucose conditions. Quercetin can also enhance the expression of SIRT1 and NMNAT; thereby, they can regulate NAD^+^ metabolite [213]. This finding suggested that inhibition of NMN accumulation may be a promising target for kidney senescence.

The combination of Dasatinib plus Quercetin (D+Q) demonstrated the ability to inhibit renal senescence and prevent the decline of renal function in both chronologically aged and transgenic aging mice [63]. This observation highlights a significant association between SCAPs and CKD. D+Q also increased α-Klotho levels in the urine, kidney, and brain of mice with an increased burden of senescent cells. In humans, D+Q elevated α-Klotho levels in the urine of patients diagnosed with idiopathic pulmonary fibrosis, a condition linked to cellular senescence [214].

The use of fisetin, another senolytic compound, reduced kidney damage in mice experiencing exacerbated atherosclerosis due to diabetes by blocking the CD36/fibrosis pathway [215,216]. Fisetin also decreased SA-β-Gal expression in senescent tubular renal cells and inhibited TGF-β-induced proliferation of renal fibroblasts [217]. In a murine model of lupus nephritis, fisetin reduced proteinuria scores and led to decreased expression of p15 in tubular epithelial cells and increased the number of proliferative Ki-67+ cells. The expression of SA-β-Gal, p53, p21, and p16 in renal tubules was reduced by fisetin in a model of cisplatin-induced murine CKD [218]. In mice with diabetes-exacerbated atherosclerosis, fisetin ameliorated the regulation of uric acid, urea, and creatinine levels, reduced morphological damages and fibrosis in the kidney, and improved glomerular function [215]. Furthermore, fisetin reduced the senescent cell burden and prevented kidney fibrosis in a telomerase-deficient progeria mouse model with elevated levels of p16 and p21 in kidney tissues [219]. This effect appeared to be mediated by induction of apoptosis in senescent cells, resulting from the inhibition of the Akt pathway through downregulation of stanniocalcin 1 (Stc1) [219]. Fisetin may also exert its senolytic effects by directly binding to and inhibiting anti-apoptotic proteins such as Bcl-xL and Bcl-2 [220,221]. These findings highlight the diverse mechanisms employed by senolytic drugs.

Other natural compounds derived from plants, such as procyanidin C1, alongside herbal extracts, demonstrate senolytic characteristics [173,222,223]. Ginsenoside, an extract sourced from ginseng, is a well-regarded anti-aging agent that hinders the aging of bone marrow rescue mesenchymal stem cells (MSC) by activating NRF2 and PI3K-Akt signaling pathways [224]. It also modulates the SASP, diminishes inflammation, sustains oxidative balance, and alleviates organ aging [225,226]. These findings suggest that targeted dietary interventions involving senolytic properties have the potential to decelerate the advancement of senescence-associated CKD.

AP20187, a synthetic dimerizer compound derived from the FK1012 system used in the INK-ATTAC transgenic model, can selectively induce apoptosis in p16-positive senescent cells by activating a caspase-based suicide mechanism in experimental mouse models [142]. In mice, targeted removal of p16 expressing senescent cells leads to a reduction in age-associated brain inflammation and stenotic kidney function and better cognitive performance [174,227]. Navitoclax (ABT-263), alongside other inhibitors of the BCL-2 family like A-1331852, A-1155463, EF24, and venetoclax, demonstrated potent senolytic impacts in aging animal models and specific types of senescent cells in laboratory investigations [175,222,228]. Particular caution may be required due to hematological side effects of some senolytics. Furthermore, inhibitors of heat shock protein 90 exhibited senolytic behavior, and radio-electric asymmetric carrier technology (REAC), a type of non-invasive therapeutic technique, was effectively used to diminish senescence in cultured stem cells [103,229].

It is important to note that certain senolytics are more specific toward particular cell types or types of senescence. Therefore, clarification is needed regarding the appropriate senolytic to use in specific contexts. To increase precision, minimize unintended impacts, and streamline the practical implementation of senolytic interventions, there is promise in utilizing strategies focused on specific organs or cells. This can be achieved through the utilization of targeted delivery technologies such as carriers, including protein-based or peptide-based systems, nanoparticles, extracellular vesicles, or alternative delivery methods. For instance, a particular approach involves attaching cytotoxic medications to antibodies that are engineered to identify the senescent cell surface marker β2-microglobulin [176]. Elevated expression of β2-microglobulin occurs through a process that is dependent on p53, indicating its correlation with senescence triggered by stress.

Another approach incorporates the activation of invariant NK T cells to enhance immune alertness and facilitate the removal of senescent cells [179]. Furthermore, senolytic impacts have been observed both in laboratory settings and within living organisms through the implementation of chimeric antigen receptor (CAR) T cells that are specifically engineered to identify the urokinase-type plasminogen activator receptor on the surface of senescent cells [180]. Moreover, encouraging outcomes have been achieved in mouse models with obesity by creating anti-aging vaccines aimed at CD153+ senescent T cells or GPNMB+ senescent endothelial cells [181].

### 8.2. Senomorphic Drugs

Senomorphic drugs have gained significant attention, with metformin standing out as a well-examined example offering diverse benefits [230]. Metformin not only reduces the occurrence of age-related diseases but may also extend the lifespans of various organisms like Caenorhabditis elegans, mice, and individuals with type 2 diabetes mellitus [182]. Metformin has also been suggested to disrupt IKK/NF-κB activation, thereby preventing the emergence of the SASP [231]. Additionally, metformin may regulate the SIRT1-p300-p53-p21 pathway to prevent endothelial senescence caused by high glucose-induced metabolic memory (metabolic memory refers to the lasting effects of transient hyperglycemia, where prior high glucose levels cause persistent damage, even after glucose normalizes) [232]. Furthermore, metformin stimulates immune-mediated removal of senescent cells and reinstates effective immune surveillance against tumors [233].

Another illustration of a senomorphic drug is ruxolitinib, a JAK inhibitor that mitigates inflammation and weakness in elderly mice by suppressing inflammatory components of the SASP [183]. Additionally, mTOR inhibitors like rapamycin display senomorphic characteristics by restraining senescence and suppressing SASP in endothelial cells [184] and fibroblasts [234] through the induction of autophagy, which reduces the accumulation of damaged cellular components. Activation of mTOR leads to mitochondrial biogenesis dependent on peroxisome proliferator-activated receptor-γ coactivator 1β, production of ROS, and persistent activation of the DDR [235]. Consequently, inhibiting mTOR may prevent cellular senescence. Similarly, the anti-fibrotic drug pirfenidone has shown renoprotective and senomorphic effects in preclinical studies by dampening profibrotic and inflammatory pathways and reducing SASP factors. More broadly, TGF-β inhibitors and anti-TNF-α therapies may also modulate SASP signaling [159,185].

Various hormones also exhibit senomorphic effects. For example, melatonin hinders the expression of SASP genes by disrupting the recruitment of poly-(ADP-ribose) polymerase 1 (PARP1) by CREB-binding protein (CBP), a sensor for DNA damage [186]. Melatonin has also been demonstrated to enhance the functionality of senescent T cells [236], alleviate mitochondrial dysfunction in the heart of a mouse model with accelerated senescence [237], and rescue MSCs from senescence triggered by uremic toxins in CKD [238]. Other hormones, such as androgens [187], estrogens [188], estradiol [189], and glucocorticoids [190], can also influence the release of inflammatory cytokines. However, care should be exercised with glucocorticoids as they can induce senescence in primary human tenocytes [190].

### 8.3. Rejuvenating Agents

Certain herbal compounds, including resveratrol [191] and other SIRT1-activating molecules, have been investigated as potential rejuvenating agents due to their ability to modulate oxidative stress, inflammation, and SASP signaling [135]. However, although these agents show promising effects in experimental models, their clinical efficacy and translational potential in CKD remain uncertain. Moreover, various activators of SIRT1 have been employed to prevent and treat senescence-related renal dysfunction [239]. Examples include SRT1460 [201], SRT1720 [202], SRT2183 [203], D-Pinitol [204], Isoliquiritigenin [205], and Rutin [240] (Table 4). Furthermore, other SIRT1 activators, such as NAD^+^, have been shown to exert beneficial effects on renal diseases; however, the timing of the treatment appears to be important [45].

Recent evidence reframes diabetic CKD as a systemic pro-ageing disorder driven by an integrated senescence–Klotho–sirtuin axis. SGLT-2 inhibitors and GLP-1 receptor agonists are now recognized not only as cardio-renal protective agents but also as modulators of molecular ageing pathways, with SGLT-2 inhibition showing indirect senolytic activity [28,29,30,31,32,33].

The expression of Klotho can be enhanced by reactivating endogenous Klotho or supplementing exogenous Klotho, leading to improvements in renal fibrosis and a reduction in senescence [206]. Strategies such as demethylation of the Klotho gene promoter, Klotho gene delivery, and inhibition of histone deacetylases have the potential to up-regulate Klotho [206]. Several drugs have been reported to increase endogenous Klotho levels [206], including intermedin, which can further alleviate senescence-related renal changes [241]. Additionally, the direct administration of exogenous soluble Klotho has proven effective in increasing circulating Klotho levels and preventing CKD in animal models. However, studies examining circulating Klotho levels and kidney function in CKD patients show conflicting results [242]. While several studies report reduced circulating or renal Klotho levels associated with CKD progression and worse renal outcomes [110,206], others have found weaker or inconsistent correlations in clinical cohorts [242]. This discrepancy may reflect differences in CKD stage, variability in methods used to measure soluble Klotho, and the possibility that Klotho deficiency may act both as a driver of cellular senescence and as a downstream consequence of chronic kidney injury [110,206]. Klotho deficiency may therefore amplify senescence pathways by increasing oxidative stress and profibrotic signaling.

Therefore, while rejuvenating strategies targeting metabolic and longevity pathways remain conceptually attractive, further clinical studies are required to clarify their therapeutic relevance in CKD.

### 8.4. Lifestyle Interventions

Lifestyle choices can either speed up or slow down aging. Insufficient sleep may accelerate aging by triggering the DDR and the release of SASP factors [243]. On the other hand, a healthy lifestyle with regular exercise and controlled calorie intake has been proposed to slow down the aging process [244,245].

Research involving animals and humans has revealed that lifelong exercise or consistent moderate physical activity among older individuals yields positive outcomes regarding immunosenescence and age-related conditions like metabolic disorders and hepatic steatosis. These benefits are attributed to the modulation of mitochondrial function, inflammation, the SASP, and the process of lipolysis [197,198,246]. Conversely, excessive caloric intake has been linked to an expedited senescence process in mice [247], while caloric restriction has been shown to reduce senescence in dogs [199] and adipose tissue in mice [248]. These findings emphasize caloric restriction’s crucial role in extending lifespan and delaying age-related chronic conditions.

Dietary interventions impact age-related well-being through changes in epigenetic patterns and shifts in the gut microbiota. Nutrients such as betaine, choline, and folate contribute to beneficial epigenetic changes, countering age-related and CKD-related alterations by targeting the methylome or chromatin. Excessive sugar intake is linked to age-related diseases through reduced microbial diversity in animal models, among many other processes [200].

Lifetime exposure to external stressors, including temperature changes, oxygen fluctuations, and poor nutrition, triggers adaptive homeostatic mechanisms [249]. Among these mechanisms are antioxidant and anti-inflammatory responses, which are activated via the NRF2-KEAP1 signaling pathway [249]. By influencing these exposures, novel approaches can be explored to prolong the phase of healthy living, combat CKD, and potentially yield favorable effects on cellular senescence. These observations suggest that the timing of senotherapeutic intervention relative to disease stage and regenerative capacity may be a critical determinant of therapeutic success.

## 9. Clinical Trials

Clinical trials examining senolytic therapies are relatively limited compared to the extensive number of animal and in vitro studies conducted so far. Translation of senotherapeutic strategies to CKD patients has been limited so far and requires careful consideration of kidney-specific physiological factors. Patients with CKD frequently exhibit impaired drug clearance and altered pharmacokinetics, which may influence the safety profile of renally cleared compounds. In addition, CKD is often accompanied by immune dysfunction, hematological abnormalities, and frailty, which may increase susceptibility to adverse effects of certain senolytic agents, particularly those targeting BCL-2 family proteins. These factors highlight the importance of evaluating senotherapeutic strategies within the specific clinical context of CKD. However, clinical studies are highly important for establishing the safety and efficacy of these therapies in humans and some have demonstrated some promising outcomes. For instance, treatment with dasatinib and quercetin has successfully reduced circulating SASP factors and the abundance of senescent cells in adipose tissue and skin of patients with diabetic kidney disease [250]. Several additional clinical trials testing the safety and efficacy of various senolytics, including D+Q and fisetin, are currently ongoing. To provide a clearer overview of the current translational landscape, selected clinical studies investigating senescence-targeting interventions are summarized in Table 5.

Interestingly, a psychosocial intervention involving horticultural therapy, which included park visits and gardening activities over six months, has been found to alleviate immunosenescence and chronic inflammation in older adults (aged 61–77 years). This intervention led to reduced levels of IL-6 and mitigated T-cell exhaustion, correlated with an improved well-being [251]. These findings highlight the importance of pursuing non-invasive and cost-effective strategies to enhance overall health and wellness.

Each of these approaches to target senescent cells, whether pharmaceutical or involving lifestyle changes, has its own set of advantages and disadvantages. Senolytics eliminate senescent cells, whereas senomorphics only suppress senescence phenotypes. Therefore, senolytics can be used in a ‘hit-and-run’ manner (e.g., once a month, or before senescent cells begin to reappear), while senomorphics would require continuous administration, potentially adding to the treatment burden of chronically ill patients. On the other hand, long-term effects of eliminating senescent cells are not fully understood, and senomorphics may therefore be less drastic than senolytics and could have fewer side effects. Further, it is possible that senolysis in certain cell types, such as post mitotic neurons or muscle cells, as well as stem cells may be detrimental as compared with a reversal strategy. Certain senolytic drugs, like navitoclax, can lead to side effects such as neutropenia, trabecular bone loss, and dysfunction of osteoprogenitor cells [252,253]. Additionally, since SASP factors play diverse physiological roles, including immunosurveillance in tumorigenesis, the potential drawbacks of senomorphic drugs might outweigh their benefits and hinder the success of clinical trials.

Moreover, the concept of antagonistic pleiotropy suggests that certain genes can offer survival advantages in the early stage of life but become detrimental later. The impact of senolytic drugs on tumorigenesis might thus depend on age, and considering the role of cellular senescence in development, the effects of senolytic drugs may vary between early and later stages of life [254]. Notably, recent research has revealed that senolytic drugs’ effectiveness is diminished in a renal artery stenosis model when premature delivery occurs. This illustrates a temporal, rather than a trait-dependent, antagonistic pleiotropy [174]. Therefore, the administration of these drugs must be carefully timed relative to both the initiating insult and the developmental stage of the patient, as these factors can influence potential outcomes.

Further exploration is needed regarding the frequency of senotherapy, considering that chronic conditions continuously generate new senescent cells. A study conducted on mice suggested that intermittent administration of senolytics could be highly effective in mitigating physical dysfunction and increasing the survival of pleiotropy [63]. Combination approaches that incorporate both senolytics and senomorphics, targeting a wide range of cell types, might prove particularly potent.

Additionally, maximizing the success of clinical trials for senotherapeutics could involve pre-screening participants to select those who exhibit evidence of cellular senescence, as senescence-based patient stratification frameworks (e.g., sendotypes) may help explain variability in therapeutic responses across biologically heterogeneous CKD populations [50]. However, to enable the selection of individuals most likely to benefit from the intervention, the development and validation of sensitive screening tools are imperative. This would ensure the identification of suitable participants who would derive the greatest advantages from the therapy.

Finally, in contrast to pharmaceutical strategies, lifestyle interventions are likely safer, but compliance and efficacy may be lower. Given the current state of knowledge, these interventions should be closely monitored by clinicians and individualized to each patient.

## 10. Conclusions

Renal senescence exhibits numerous parallels with CKD, including causative factors, mechanisms, pathological alterations, characteristics, and consequences. The reviewed literature highlights cellular senescence as a central mechanism driving both kidney aging and CKD. Nevertheless, determining whether renal senescence serves as a catalyst or an outcome of CKD remains challenging, as current evidence suggests a bidirectional relationship in which kidney injury promotes senescence, while senescent cells further promote inflammation and fibrosis, thereby contributing to disease progression [50].

Furthermore, current methods for evaluating senescence vary by condition and organ, emphasizing the need for highly sensitive and specific non-invasive techniques. Since many inflammatory factors are components of the SASP, their expression alone is insufficient to identify cellular senescence accurately. Urinary exosomes bearing specific markers have shown potential in detecting kidney senescence [255], but standardized methods for their collection and characterization are still required. In parallel, AI and ML are emerging as powerful tools to enhance senescence assessment by integrating multi-omics data, nuclear morphology imaging, and clinical biomarkers. Future progress will depend on integrating AI-derived senescence metrics with longitudinal clinical data and multi-center validation frameworks.

For clinical trials evaluating senotherapeutic potential, it will be vital to establish clinical endpoints and indicators capable of measuring the therapeutic success of senotherapeutics. Potential outcome measures may include biomarkers of renal fibrosis, circulating or urinary senescence-associated factors such as urinary clusterin, extracellular vesicle signatures, and systemic inflammatory markers, which may complement traditional clinical measures such as eGFR. In the future, novel senescence biomarkers combined with AI- driven models may help guide the identification and management of patients most likely to benefit from interventions targeting cellular senescence.

## Figures and Tables

**Table 2:** Applications of AI/ML in Senescence and CKD Research.

Purpose	AI/ML Approach	Outcome	Ref.
Omics-based biomarker discovery and patient stratification in CKD	SVM, Random Forest, LASSO, multi-algorithm ML	Identified senescence-associated hub genes (e.g., LIMA1, ZFP36, PTEN) Validated circulating biomarkers (CKAP4, PTX3) Developed senescence-related risk signatures to stratify CKD/AKI patients	[87,88,89]
Computer Vision–Based AI for nuclear morphology and tissue senescence detection	Random Forest, Classification Trees, Deep Neural Networks	Generated tissue senescence scores Predicted senescent cells in vitro and in tissue micrographs Applied deep learning to PBMC nuclei to assess environmental/altitude effects on aging	[90,91,92,93]

**Table 4:** Senotherapeutic strategies to address issues related to senescence in kidney. These approaches differ in translational readiness and may depend on CKD stage and safety considerations, and timing of intervention.

Senotherapeutic Method	Group	Examples	Ref.
Senolytic agents	SCAP-modulation	Quercetin, fisetin, AP20187, navitoclax, A-1331852, A1155463, EF24 and venetoclax, antibody engineered toxic drugs, ginsenoside	[18,172,173,174,175,176,177,178]
	Immune treatments	Chimeric antigen receptor T cells, activator of invariant natural killer T cells, vaccines	[179,180,181]
Senomorphic agents	SASP modulators	Metformin, ruxolitinib, rapamycin, melatonin, androgen, estrogen, estradiol, glucocorticoids, Pirfenidone	[182,183,184,185,186,187,188,189,190,191]
Stem cells	Stem cells		[192,193,194]
	Extracellular vesicles derived from stem cells	MSC-derived extracellular vesicles, antler Stem cell-derived extracellular vesicles	[195,196]
Non-pharmacological interventions	Lifestyle interventions	Moderate regular exercise, Nourishing diet, calorie restriction	[197,198,199,200]
Rejuvenating agents	Senescence modulators	Resveratrol, SRT1460, SRT1720, SRT2183, Isoliquiritigenin, Rutin, Klotho, PPAR-γ agonists, D-Pinitol	[98,163,201,202,203,204,205,206,207]

SASP: Senescence-Associated Secretory Phenotype; SCAP: senescent cell anti-apoptotic pathways; MSC: Mesenchymal Stem Cells.

**Table 5:** Selected clinical trials investigating senescence-targeting interventions in human disease.

Intervention	Patient Population	Clinical Trial	Primary Endpoints	Senescence Readouts	Clinical Trial ID
Dasatinib + Quercetin	Diabetic kidney disease	Phase I	Safety and feasibility; changes in senescent cell burden	Reduction in p16INK4A+ and p21CIP1+ cells, decreased SA-β-gal activity, reduced circulating SASP factors (IL-1α, IL-6, MMP-9, MMP-12)	NCT02848131
	idiopathic pulmonary fibrosis	Phase I	Physical performance and safety	Circulating SASP markers	NCT02874989
	Early Alzheimer’s disease	Phase II	Safety and feasibility of senolytic therapy	Inflammatory and aging-related biomarkers associated with SASP	NCT04063124
Fisetin	Older adults with frailty	Phase II	Improvement in physical function and frailty-related outcomes	Circulating inflammatory markers and SASP-associated cytokines (e.g., IL-6, TNF-α)	NCT04313634
Fisetin	Older adults with multimorbidity	Phase I/II	Pharmacokinetics, safety, and inflammation	Circulating levels of inflammatory, SASP, aging- and senescence-related biomarkers (e.g., suPAR)	NCT06431932
UBX1325 (BCL-xL inhibitor)	diabetic macular edema/diabetic eye disease	Phase II	Improvement in visual acuity and retinal thickness	Indirect assessment through functional and inflammatory biomarkers associated with senescent retinal cells	NCT04857996
UBX0101	knee osteoarthritis	Phase II	Pain reduction and joint function	SASP-related inflammatory biomarkers	NCT04129944

## Data Availability

No new data were created or analyzed in this study. Data sharing is not applicable to this article.

## Abbreviations

The following abbreviations are used in this manuscript:

α-SMA	Alpha-Smooth Muscle Actin
ADR	Adriamycin (Doxorubicin)
ADR model	Adriamycin-Induced Nephropathy Model
AI	Artificial Intelligence
AKI	Acute Kidney Injury
AMPK	AMP-Activated Protein Kinase
ATM	Ataxia Telangiectasia Mutated
BCL-2	B-Cell Lymphoma 2
CB2	Cannabinoid Receptor Type 2
CBP	CREB-Binding Protein
CD153	Cluster of Differentiation 153
CD38	Cluster of Differentiation 38
CDK	Cyclin-Dependent Kinase
CDKN1A	Cyclin-Dependent Kinase Inhibitor 1A
CKAP4	Cytoskeleton-Associated Protein 4
CKB	Creatine Kinase B
CKD	Chronic Kidney Disease
CLCA1	Chloride Channel Accessory 1
DDR	DNA Damage Response
DNA	Deoxyribonucleic Acid
ECM	Extracellular Matrix
eGFR	Estimated Glomerular Filtration Rate
EFNB1	Ephrin-B1
EFNB3	Ephrin-B3
EMT	Epithelial–Mesenchymal Transition
ERK	Extracellular Signal–Regulated Kinase
ESRD	End-Stage Renal Disease
FGF21	Fibroblast Growth Factor 21
FGF23	Fibroblast Growth Factor 23
FOS	Fos Proto-Oncogene, AP-1 Transcription Factor Subunit
FOXO4	Forkhead Box O4
FSGS	Focal Segmental Glomerulosclerosis
γH2AX	Phosphorylated Histone H2AX
G2/M	Gap 2/Mitosis Phase
GPNMB	Glycoprotein Non-Metastatic Melanoma Protein B
H2AX	Histone H2AX
HIV	Human Immunodeficiency Virus
HSP90	Heat Shock Protein 90
IGFBP6	Insulin-Like Growth Factor Binding Protein 6
IKKβ	IκB Kinase Beta
IL-1α	Interleukin-1 Alpha
IgAN	IgA Nephropathy
INK-ATTAC	INK4a Apoptosis Through Targeted Activation of Caspase (Transgenic Mouse Model)
IRI	Ischemia–Reperfusion Injury
JAK	Janus Kinase
JNK	c-Jun N-Terminal Kinase
KAT7	Lysine Acetyltransferase 7
KEAP1	Kelch-Like ECH-Associated Protein 1
Ki-67	Marker of Cellular Proliferation
Klotho	α-Klotho Protein
LASSO	Least Absolute Shrinkage and Selection Operator
LIMA1	LIM Domain and Actin Binding 1
LMNB1	Lamin B1
MAPK	Mitogen-Activated Protein Kinase
MCD	Minimal Change Disease
MCP-1	Monocyte Chemoattractant Protein 1 (CCL2)
MDM2	Murine Double Minute 2
ML	Machine Learning
MN	Membranous Nephropathy
MSCs	Mesenchymal Stem Cells
mTOR	Mechanistic Target of Rapamycin
MYD88	Myeloid Differentiation Primary Response 88
NAD+	Nicotinamide Adenine Dinucleotide
NAMPT	Nicotinamide Phosphoribosyltransferase
NF-κB	Nuclear Factor Kappa B
NIH	National Institutes of Health
NMNAT	Nicotinamide Mononucleotide Adenylyltransferase
NRF2	Nuclear Factor Erythroid 2–Related Factor 2
nsMRA	Non-steroidal Mineralocorticoid Receptor Antagonist
p15^INK4b	Cyclin-Dependent Kinase Inhibitor 2B
p16^INK4a	Cyclin-Dependent Kinase Inhibitor 2A
p19^Arf	Alternative Reading Frame Protein Encoded by Cdkn2a (Mouse)
p21^CIP1	Cyclin-Dependent Kinase Inhibitor 1A
p27^Kip1	Cyclin-Dependent Kinase Inhibitor 1B
p38 MAPK	p38 Mitogen-Activated Protein Kinase
p53	Tumor Protein 53
p65	RELA (NF-κB Subunit)
PARP1	Poly (ADP-Ribose) Polymerase 1
PBMC	Peripheral Blood Mononuclear Cells
PCNA	Proliferating Cell Nuclear Antigen
PI3K	Phosphoinositide 3-Kinase
PINK1	PTEN-Induced Kinase 1
PTEN	Phosphatase and Tensin Homolog
PTX3	Pentraxin 3
RAAS	Renin–Angiotensin–Aldosterone System
RB	Retinoblastoma Protein
ROS	Reactive Oxygen Species
SADF	Senescence-Associated Distension of Satellites
SAHF	Senescence-Associated Heterochromatin Foci
SA-β-Gal	Senescence-Associated β-Galactosidase
SASP	Senescence-Associated Secretory Phenotype
SCAPs	Senescent Cell Anti-Apoptotic Pathways
SIPS	Stress-Induced Premature Senescence
SIRT1	Sirtuin 1
STAT3	Signal Transducer and Activator of Transcription 3
SVM	Support Vector Machine
TGF-β	Transforming Growth Factor Beta
TMEM16A	Transmembrane Protein 16A
TNF	Tumor Necrosis Factor
UUO	Unilateral Ureteral Obstruction
Wnt9a	Wnt Family Member 9A
Wnt/β-catenin	Wnt/Beta-Catenin Signaling Pathway
ZFP36	Zinc Finger Protein 36
